# Energy expenditure during nutritional rehabilitation: a scoping review to investigate hypermetabolism in individuals with anorexia nervosa

**DOI:** 10.1186/s40337-024-01019-7

**Published:** 2024-05-21

**Authors:** Kylie K. Reed, Ava E. Silverman, Afrouz Abbaspour, Kyle S. Burger, Cynthia M. Bulik, Ian M. Carroll

**Affiliations:** 1 Department of Nutrition, University of North Carolina at Chapel Hill, Chapel Hill, NC USA; 2 Department of Psychiatry, University of North Carolina at Chapel Hill, Chapel Hill, NC USA; 3 Smith College, Northampton, MA USA; 4 Department of Medical Epidemiology and Biostatistics, Karolinska Institutet, Stockholm, Sweden

**Keywords:** Anorexia nervosa, Resting energy expenditure, Weight restoration, Metabolism, Hypermetabolism, Eating disorders, Scoping review

## Abstract

**Background:**

Weight gain and nutritional rehabilitation are essential first steps to achieve medical stabilization in anorexia nervosa, and frequent resistance to weight gain requires patients to consume high kilocalorie loads. Adaptive hypometabolism is common when patients begin treatment, and rebound hypermetabolism is suspected to be a significant barrier to weight gain. The aim of this review was to summarize existing data describing metabolic changes in anorexia nervosa during weight restoration. The reported findings challenge current hypotheses of weight gain resistance and highlight key areas for future research.

**Methods:**

Using scoping review guidelines, three databases were searched for studies investigating metabolic changes in anorexia nervosa before and after renourishment. Two reviewers systematically screened the titles and abstracts of 447 articles, and full-text versions of 106 studies were assessed for eligibility. A total of 36 studies were included for review. Data regarding the study description, sample population (including age, weight, BMI, duration of treatment, and caloric intake), and metabolic variable descriptions were extracted.

**Results:**

Female patients with anorexia nervosa from studies across 13 countries were included. Across the studies, average BMI increased from 13.7 kg/m^2^ at admission to 17.57 kg/m^2^. Patients presented to treatment with clinically reduced energy expenditure levels. After varying levels of nutritional rehabilitation and weight restoration, measured energy expenditure increased significantly in 76% of the studies. Energy expenditure values at the second timepoint increased to the standard range for normal weight female teenagers and adults. Despite these increases, the studies do not indicate the presence of a hypermetabolic state during renourishment. Additionally, all studies including both measured and predicted energy expenditure reported that predicted energy expenditure overestimated measured values.

**Conclusion:**

This study provides a detailed evaluation of the literature investigating energy expenditure and metabolic rate in patients with anorexia nervosa before and following a period of renourishment. The findings from this review identify important gaps in the current beliefs of energy expenditure in anorexia nervosa and highlight a need for further exploration of metabolic alterations during weight restoration.

## Abstract (plain language summary)

Nutritional rehabilitation and weight restoration are two primary goals of anorexia nervosa treatment that pose significant physiological and psychological challenges for patients. Patients often require high caloric loads to continue an adequate weight gain trajectory, but the underlying cause of weight gain resistance remains unknown. We completed a scoping review of research into energy expenditure and metabolic rate during treatment. Our search identified 447 relevant articles from academic databases, and 106 were deemed eligible after screening. We extracted data, including sample characteristics, kilocalorie intake, energy expenditure, and treatment information, from 36 studies. When individuals arrived for treatment, their energy expenditure was lower than that of individuals without an eating disorder due to the prolonged state of nutrient deprivation. After varying amounts of time and kilocalorie intake, most studies reported significant increases in energy expenditure. However, energy expenditure after a period of renourishment did not indicate an overactive metabolism (i.e., “hypermetabolism”). Funders should consider supporting exploration of additional factors that may be functioning as barriers to weight gain during treatment, in pursuit of making treatment more efficient and long-lasting. Additionally, future research describing metabolism in anorexia nervosa should provide more consistent methodologies, robust statical testing, and comprehensive reporting of dietary intake.

## Introduction

### Background

Anorexia nervosa (AN), a severe psychiatric disorder characterized by extreme weight dysregulation, carries one of the highest mortality rates of all psychiatric illnesses [1, 2]. Affecting individuals of all ages, ancestries, and genders, AN has an estimated lifetime prevalence of up to 4% in females and 0.3% in males [3]. Within the AN diagnosis, patients may be classified as restricting or binge eating/purging type, and nearly 75% of patients with AN report a comorbid lifetime mood or anxiety disorder [2, 4]. Somatic symptoms associated with AN, although varying with severity and stage of illness, affect nearly every organ system in the body due to starvation or binge eating and/or purging behaviors [5]. AN presentation involves a complex interplay between biological, psychological, genetic and environmental factors, and remediating the consequences of severe caloric restriction are only one component of the current treatment paradigm [6].

To achieve medical stabilization, weight gain and nutritional rehabilitation (also referred to as clinical refeeding, renourishment, or weight restoration) are the first and essential goals of treatment. The process of therapeutic renourishment can be both physically and psychologically uncomfortable and relapse is high [7, 8]—highlighting the need for novel, safe, and effective therapeutic approaches. Full recovery is more common in adolescents than in adults [9], and on average, adult patients require 5–6 years of treatment until achieving remission [10]. Throughout the course of the illness, it is common for both adolescent and adult patients to undergo several cycles of therapeutic renourishment followed by loss of restored weight before achieving lasting recovery. One potential explanation for the lack of efficacy of current renourishment strategies is the need to consume a sustained high kilocalorie (kcal) diet, which is psychologically (due to fear of weight gain) and physically (due to discomfort, pain, and gastrointestinal complications) challenging to patients and can lead to premature discontinuation of treatment and jeopardize long-term recovery [11, 12]. As metabolic output forms the basis for caloric intake prescription, meticulous monitoring of the metabolic profile in individuals receiving treatment for AN is crucial for achieving successful renourishment. Research to advance the nutritional component of AN treatment protocols, however, lacks an updated and comprehensive characterization of metabolic changes in patients with AN.

### Metabolic adaptations in anorexia nervosa

Metabolism is a series of chemical reactions by which calories consumed are converted (catabolized) into usable energy for healthy body function. Metabolic rate is often measured by indirect calorimetry and predicted using calculations including the Harris-Benedict and Schebendach equations [13]. Previous work has highlighted that these equations may not accurately predict metabolic rate in AN [14], as classical energy expenditure estimation based on age, height, weight, and sex fails to consider the metabolic profile in a disease state [15]. For AN, therapeutic renourishment plans are developed on the foundation of metabolic requirements and tailored to each individual patient. The current standard of care varies widely across treatment facilities, with some recommendations starting at 10 kilocalorie (kcal)/kg/day with increases of 5 kcal/kg/day [16] and others starting at 30–40 kcal/kg/day with increases up to 70–100 kcal/kg/day to promote faster weight gain [17]. Recent studies have highlighted the efficacy of higher-calorie refeeding, both in restoring medical stability more quickly and reducing overall hospital charges per participant [18]. Importantly, patients with AN in a chronic state of starvation can experience significant decreases in metabolic rate to compensate for the lack of energy intake [19]. These *hypo*metabolic states are typically attributed to reduced protein turnover (i.e., reduced renewal and replacement of protein) and ATP supply pathways, providing the body with significant energy savings [20].

Adaptive hypometabolism prior to renourishment is common and well-documented [21, 22]. Less clear is whether rebound *hyper*metabolism (either from a hypo- or normo-metabolic baseline) occurs as commonly during nutritional rehabilitation. When weight gain stalls during inpatient or residential treatment, two common hypotheses are typically considered: (i) the patient is using exercise or other methods to inhibit weight gain, or (ii) the patient’s metabolic rate has shifted to a *hyper*metabolic state [23]. Hypermetabolic states are characterized by increased physiological responses including heart rate, blood pressure, body temperature, and protein and lipid catabolism, leading to excessive resting energy expenditure [24]. Hypermetabolism has been well described in circumstances of both chronic and acute stress (e.g., trauma [25], severe burn injury [26], and COVID-19 [27]), so it is conceivable that physiological stress from AN progression, in addition to other common AN complications such as chronically elevated cortisol [28] and difficulty sleeping [29], may also contribute to increased cellular metabolism during treatment. Anecdotal reports posit that increases in metabolic rate may be an important barrier to weight gain and weight maintenance in AN [23], and limited evidence reports a shift towards hypermetabolism during AN treatment [30, 31]. Proposed explanations for accelerated energy expenditure during nutritional rehabilitation include the occurrence of night sweats, irregular and/or elevated heart rate, and nervous system dysfunction [23].

As nutritional rehabilitation programs are centered around metabolic rate estimations, it is essential for providers to have an accurate depiction of the metabolic setting in each individual patient. Caloric initiation standards are based on basal metabolic rate (BMR); however, BMR is infrequently remeasured once renourishment begins, leaving dietetic teams to increase caloric load based on careful observation of weight gain, food intake, and physical activity on the unit [14]. Additionally, it is likely that studies investigating metabolism during AN treatment employ equations that were not developed for application to low body weight individuals [32]. These challenges highlight the need for comprehensive documentation of metabolic changes before, during, and after renourishment, rather than relying on a cross-sectional metabolic assessment upon hospital admission.

In an effort to consolidate and evaluate existing knowledge about metabolic changes during renourishment in individuals with AN, we conducted a scoping review of energy expenditure (EE) during nutritional rehabilitation. We anticipated finding consistent reports of moderate increases in energy expenditure after renourishment and aimed to aggregate existing knowledge on the commonly referred to “hypermetabolic state” that occurs during renourishment.

## Methods

### Search strategy

A scoping review search was performed in June and July 2023 through Covidence using PRISMA guidelines. Searches were conducted in PubMed, SCOPUS, and the Cumulated Index in Nursing and Allied Health Literature (CINHAL). Search terms included (“Energy Metabolism” [Mesh] OR “Energy Expenditure” OR “energy expenditures” OR “hypermetabolism” OR “hypermetabolic” OR “metabolic rate” OR “basal metabolism” OR “metabolic rates” OR “energy metabolism”) AND (“Anorexia Nervosa” [Mesh] OR “anorexia nervosa”). Medical Subject Headings (i.e., MeSH) assigned to files in PubMed were used to retrieve all records on the relevant subject (metabolism and AN) in a subjective manner, regardless of the vocabulary utilized by the author.

### Inclusion and exclusion criteria

Studies and articles (including peer reviewed conference abstracts) published between 1980 and 2022 that investigated metabolic changes in patients with AN before (T1) and after (or during) renourishment (T2) were eligible for analysis. Relevant studies that reported any metabolic variable outcome, including resting energy expenditure (REE), total energy expenditure (TEE), basal metabolic rate (BMR), and resting metabolic rate (RMR) and recorded at least two time points (e.g., admission and discharge from treatment unit, baseline and follow-up from treatment, independent groups of active cases and recovered patients, etc.) were included. The following exclusion criteria were applied: (1) cross-sectional studies reporting only one metabolic timepoint; (2) case studies; (3) qualitative studies; (4) animal studies; (5) studies of individuals with other eating disorders; (6) presentations, dissertations, theses, book chapters, and other technical documentation.

### Study selection and data extraction

**Fig. 1:**
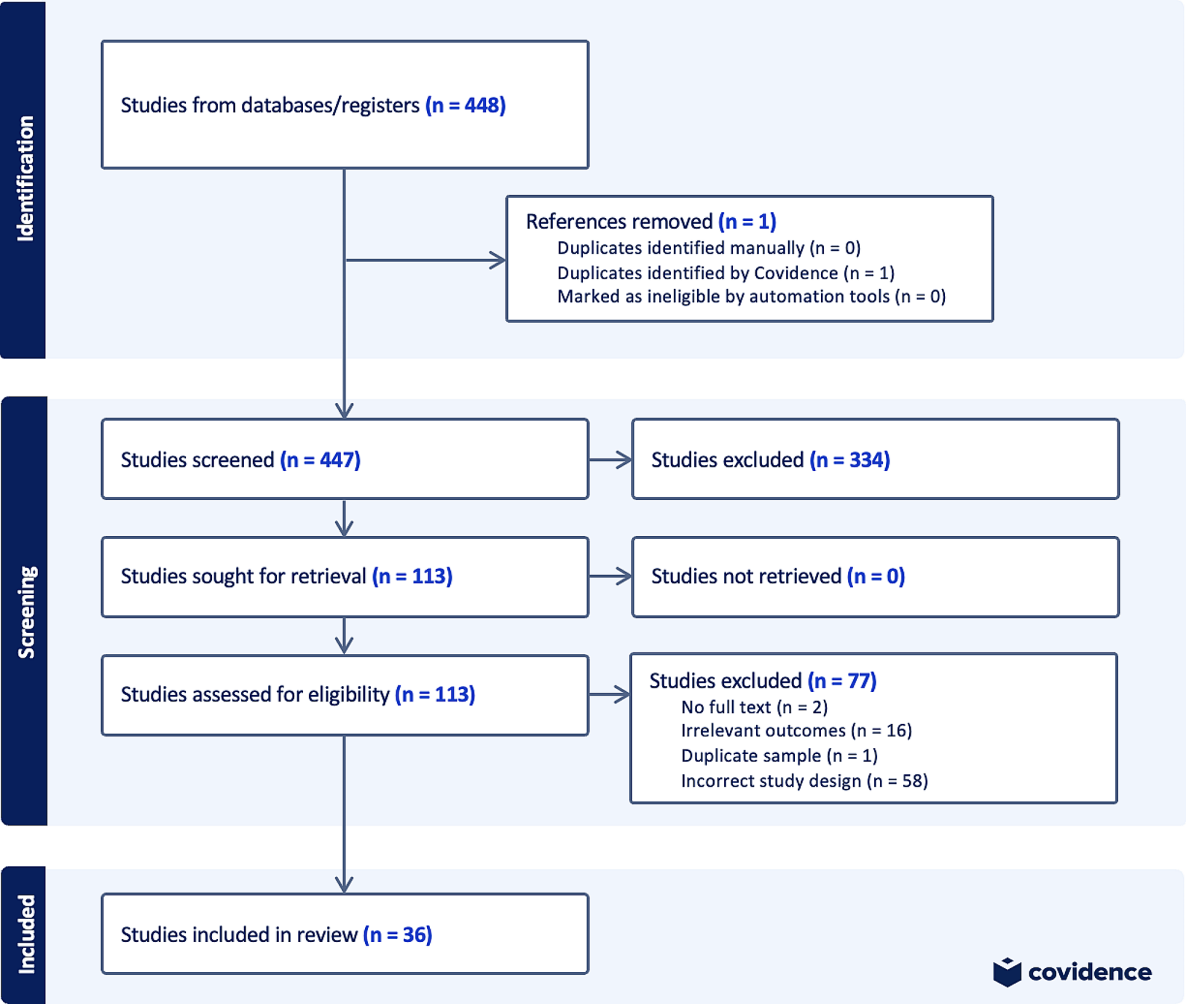
PRISMA flowchart depicting article identification, screening, and inclusion

A flowchart was created to document general article screening progress in accordance with PRISMA guidelines (Fig. 1). K.R. and A.S. used Covidence to systematically assess articles using titles, abstracts, and full text copies. After the initial search, 448 studies were imported for screening, one of which was a duplicate. Of the 447 studies screened, 334 studies were deemed irrelevant based on title and/or abstract screening according to inclusion and exclusion criteria. The remaining 106 full-text studies were assessed for eligibility, and 70 were excluded due to study design (*n* = 55) or outcomes (*n* = 15) not of relevance for this review. A total of 36 studies were included (Fig. 1).

A Covidence-generated template was used to extract the following information: (1) Author and year of publication; (2) country where the study was conducted; (3) aim of the study; (4) inclusion and exclusion criteria; (5) population characteristics (including age, weight, BMI, duration of treatment, and caloric intake when available); (6) metabolic variable descriptions, numerical results, and correction factors. All study participants met diagnostic criteria for AN according to the DSM-III, DSM-IV, or DSM-5, depending on time of publication. Means and standard deviations were evaluated for each numeric variable when available. Metabolic variables (REE, TEE, RMR, and BMR) were standardized to kcal/day, and the longest duration from follow-up reported in each study was recorded for this review to maintain consistency throughout data extraction. Means of clinical characteristics and percent change in EE values from baseline to follow-up were calculated.

## RESULTS

### Study characteristics

The included studies evaluated female patients with AN in Australia (*n* = 2), Brazil (*n* = 1), Canada (*n* = 2), Czech Republic (*n* = 2), France (*n* = 4), Germany (*n* = 3), Israel (*n* = 2), Italy (*n* = 4), Japan (*n* = 2), Spain (*n* = 4), Sweden (*n* = 1), Switzerland (*n* = 1), and USA (*n* = 8) (Table 1). Studies were published between 1984 and 2020. A non-eating disorder (non-ED) control group was included in 55% of studies (*n* = 20). In addition to healthy female controls, three studies included “refed”, “rehabilitated”, or “recovered” patients with AN [33–35]; one study included a comparison group of weight-recovered females with an acute episode of AN in the last 10 years [36]; and one study included an additional comparison of “malnourished, dying patients” with other diseases [37]. Four of the studies [34, 35, 38, 39] recruited from outpatient treatment facilities, whereas the majority of included patients with AN were receiving inpatient treatment. All studies included only female participants across both AN and control groups, and only data on patients with AN were extracted.

The studies reported varying renourishment protocols, and energy intake ranged from 753 [40] to 3264 [41] kcal at baseline (T1) and 1658 [42] to 3600 [43] kcal at follow-up (T2). With the exception of Pettersson 2016 (-19.67% change in kcal) [41] and Pauly 2000 (0.00% percent change in kcal) [42], studies reported a higher kcal intake at the second timepoint (ranging from + 11.82% [44] to + 217.76% [45]). When the first measurement was obtained, the study participants had an average BMI of 13.7 kg/m^2^. At the T2 measurement and an average of 67 days of renourishment, the patients gained significant weight, had an average BMI of 17.57 kg/m^2^. Of note, the mean BMI at T2 falls outside of the recommended range for BMI (18.5–25 kg/m^2^).

**Table 1:** Study characteristics

Study	Country	*N*	Age	Days of Treatment	Kilocalorie Intake T1	Kilocalorie Intake T2	Kilocalorie Percent Change	Weight (kg) T1	Weight (kg) T2	Weight% Change	BMI T1	BMI T2
**Restricting Type**												
Melchior 1989	France	11	21.4	28	852			35.2	43.6	23.86	13.26	16.46
Scalfi 1993	Italy	19 malnourished 10 refed	22.4	n/a				37.2	52.6	41.40%	14.6	20.9
Platte 1994	Germany	6 inpatient 6 recovered	27.5	n/a				43.1	62.9	45.94%	15.2	21.2
Moukaddem 1997	France	11	23.6	7	783	2054	162.32%	33.7	35.1	4.15%	12.9	13.5
Van Wymelbeke 2004	France	87	23.4	75	822	2612	217.76%	35.8	45.4	26.82%	13.3	16.9
Yoshida 2006	Japan	9	21.4		1022	1667	63.11%				12.9	13.3
Cuerda 2007	Spain	11*	14.7	54	1000–1600	2000–2500+		40.4	45.3	14.39%	15.1	17.1
**Restricting and Binge eating/Purging**												
Agüera 2015	Spain	ANR:70 BP: 48	ANR:25.4 BP: 28.3	75	1200	2300	91.67%				ANR:16.7 BP: 17.2	ANR:17.7 BP: 18.6
**Unspecified Type**												
Dempsey 1984	USA	4	25.5	63				29.9	40.8	36.45%	11.7	15.9
Vaisman 1991	Canada	18*	15.8	61	1645	2223	35.14%	38.2	44.5	16.49%	15	17.5
Obarzanek 1994	USA	10	23.3	153	1105	3216	191.04%	36.6	47.4	29.51%	14.1	18.2
Krahn 1993	USA	10	19–38	44	1200	3600	200.00%	39.1	45.6	16.62%		
Pichard 1996	Switzerland	9	17.1	70	1267	2422	91.16%	38.1	44.1	15.75%	13.7	15.9
Schebendach 1997	USA	50*	16.3	42	1449	3018	108.28%	37.3			14.7	
Svobodová 1999	Czech Republic	9									14.5	15.6
Rigaud 2000	France	16	25.2	28	840	1680	100.00%	38.1			13.6	
Pagliato 2000	Italy	32 AD 17 DIS	23.5					34.4	39.3	14.24%	13.2	15.2
Pauly 2000	Canada	21	25	7	1658	1658	0.00				15.5	16.3
Polito 2000	Italy	16 active 14 rehabilitated	25	n/a				41.6	51.5	23.80%	15.5	20
Russell 2001	Australia	34 AD 18 DIS	20.9		1000–1500	2500–4000		41.4	49.9	20.53%	15.6	19.0
Satoh 2003	Japan	10	14	56	753	2029	169.46%	24.6				
Vaisman 2004	Israel	7	15–24					38.9	44.7	14.91%		
Cuerda Compés 2005	Spain	21 AD 9 DIS	17	55				39.8	45.7	14.82%	15.5	17.8
Haas 2005	Germany	19*	25	84	1802	2015	11.82%	41.9	50.9	21.48%	14.6	17.7
Onur 2005	Germany	28 AD* 17 DIS*	25	42	1821	2335	28.23%	42.9	49.6	15.62%	15.1	17.5
Winter 2005	USA	8		28							12.5	15.3
Dragani 2006	Italy	27		333				45.2	47.2	4.42%	17.3	18.1
Forman-Hoffman 2006	USA	7	24.3	21		3500		37.3			13.7	15.2
Konrad 2007	USA	10	31	56	2187	3217	47.10%	41.7	48.2	15.59%	15.4	
Dostálová 2010	Czech Republic	16	24.9	60							15.7	17.5
Haas 2010	Australia	37	14.6	43				42.7	47.7	11.71%	16	17.7
Sum 2011	USA	36*	23.4	66	1800	2600	44.44%	41.5	53.8	29.64%	15.8	20.4
Soto-Célix 2013	Spain	31	30.5	42	42.6 kcal/kg	51.1 kcal/kg		41.5	46.4	11.81%	15.9	17.8
Pettersson 2016	Sweden	21	19.9	84	3264	2622	-19.67%	44.4	54.2	22.07%	15.5	19.0
Kochavi 2020	Israel	35	15.41	120	1100	3000–3500		38.7	50.3	29.97%	15.2	19.9
DosReis 2022	Brazil	9 active 9 recovered	23	n/a				39.4	51.4	30.46%	15.9	20.3

Studies marked with * have available energy expenditure data (Table 2) on a smaller subset of participants than are described in Table 1. T1: initial measurement; T2: follow up measurement; BMI: body mass index; AD: admission; DIS: discharge. ANR: Restricting Type; BP: Binge-eating/purging type; n/a: not applicable

### Measured energy expenditure

Measured energy expenditure is reported in Table 2. The energy expenditure measurement with the longest duration from baseline was extracted as the second timepoint (T2). Of the included studies, 76% (*n* = 28) reported statistically significant increases in energy expenditure from baseline (T1) to follow-up (T2) timepoints. In contrast, five studies reported no significant differences [36, 46–48]. An additional four studies did not report numerical summaries of relevant statistical tests [43, 49–51]. On average, the studies reported a 24.99% change in EE from baseline to follow-up. Cuerda Compés 2005 reported the smallest percent change of 5.18% [52], and the EE values reported in Obarzanek 1994 showed the largest percent change of 62.80% [53].

**Table 2:** Measured energy expenditure

Study	Baseline EE (kcal/day)	Discharge EE (kcal/day)	Statistical Significance	Percent Change from Baseline	Other observations
**Resting Energy Expenditure (REE)**					
**Restricting**					
Melchior 1989	831	1017	*P <* 0.05	22.38%	When expressed per kg of LBM, REE was unchanged after dietary repletion and weight gain. After weight gain, REE was correlated with LBM (*r* = 0.69, *P* < 0.02). When expressed as % basal values, the increase of REE was positively correlated with LBM (*r* = 0.64, *P* < 0.05).
Scalfi 1993	836.28	1181.17	*P <* 0.01	41.24%	EE remained significantly lower even after adjustment for body composition (FFM and FM) or body weight.
Moukaddem 1997	1031.55	1104.69	*P* < 0.021	7.09%	When the increase in body weight and LBM was taken into account, one week of refeeding did not modify REE.
Van Vymelbeke 2004	918.74	1397.80	*P* < 0.001	52.14%	The ratio of REE to FFM was significantly related to energy intake (*P <* 0.01), anxiety (*P <* 0.01), abdominal pain (*P <* 0.05), and depressive mood (*P <* 0.05). The ratio also increased significantly with physical activity (*P <* 0.01) and cigarette smoking (*P <* 0.02). This rise in REE leveled off after recovery from AN.
Yoshida 2006	761.9	934.0	*P* = 0.000 paired t-test	22.59%	EE remained significantly lower at baseline even after adjustment for LBM.
Cuerda 2007	1106	1241	*P* = 0.011 Wilcoxon	12.21%	REE (kcal/day) significantly increased during hospitalization. However, the increase in REE/kg FFM did not reach statistical significance.
**Unspecified**					
Dempsey 1984	766	1240	Not reported	61.88%	
Vaisman 1991	831.98	1108.99	*P* < 0.025	33.30%	EE remained significantly lower at admission even when expressed per kg of body weight or unit of LBM. No significant correlation was found between total energy or protein intake and total REE or REE per kg of LBM.
Krahn 1993	1166	1769	Not reported	51.72%	
Schebendach 1997	895.2	1123.8	*P* < 0.001	25.54%	
Svobodovå 1999	1188.58	1257.89	*n.s.*	5.83%	
Pagliato 2000	857	1100	*P =* 0.0028	28.35%	A positive correlation was found at T1 (*r* = 0.62) between lean body mass and REE, whereas at T0, the correlation was not significant.
Pauly 2000	1086	1193	*P =* 0.0037	9.85%	
Rigaud 2000	918.74	1233.27	*P <* 0.01	34.23%	
Russell 2001	1038	1211	*P <* 0.001	16.67%	
Satoh 2003	830	1230	*P <* 0.01	48.19%	
Vaisman 2004	882.4	1006.7	*P <* 0.01	14.09%	
Cuerda Compés 2005	1165.2	1225.5	*P <* 0.05	5.18%	
Haas 2005	989.48	1123.33	*P <* 0.05	13.53%	No significant correlation between serum leptin and REE (tested within AN patients only).
Onur 2005	1027.72	1123.33	*P <* 0.01 Wilcoxon	9.30%	There was a highly significant association between weight gain-induced changes in Triiodothyronine and changes in adjusted REE (r = 0.78, P < 0.001, based on Pearson’s correlation). An increase in plasma Triiodothyronine concentrations of 1.8 pmol/l could explain an increase in REE of 0.6 MJ/day (that is, a 32% increase in Triiodothyronine was associated with a 13% increase in REE).
Winter 2005	1058	1133	*n.s.*	7.09%	
Dragani 2006	882.74	992.38	*P <* 0.03	12.42%	Pre-/post-rehabilitation difference: *P* < 0.005. REE/day shows significant positive correlation with BMI, weight, arm muscle area, and arm fat area (*P* < 0.001). Change in REE/day significantly correlates with change in BMI (*P* < 0.01).
Haas 2010	995	1158	*P* < 0.001	16.38%	Positive association between leptin and REE_LTM_ in AN (r^2^ = 0.14, P = 0.001). After weight gain, the slope of the regression line was less steep when compared with AN before weight gain.
Dostálová 2010	1060.0	1176.3	*P <* 0.05	10.97%	
Sum 2011	1087	1378	*P <* 0.05	26.77%	
Soto-Célix 2013	1023	1128	*P =* 0.08	10.26%	
Kochavi 2020	972.60	1255.40	Not reported	29.08%	Admission measured REE was positively correlated with admission weight and BMI, as well as with longer duration of illness. Measured discharge REE was positively correlated with duration of illness. Greater changes in measured REE were correlated with lower admission weight, BMI, and weight/height ratio, and with greater increase in weight from admission to discharge. No significant correlations were found between any of the REE measurements and thyroid hormone and cortisol levels at admission and discharge or the change in their levels from admission to discharge.
**Total Energy Expenditure (TEE)**					
**Restricting**					
Platte 1994	1946	2602	*n.s.*	33.71%	
**Unspecified**					
Pettersson 2016	1568	2034	*P <* 0.0001	29.72%	
**Resting Metabolic Rate (RMR)**					
**Restricting**					
Platte 1994	1171	1330	*n.s.*	13.58%	Significant correlations between RMR and LBM were found in the weight-regained group (*r* = 0.84; *P* < 0.05), but not in the acute group (*r* = 0.39; ns).
**Unspecified**					
Obarzanek 1994	742	1208	*P* < 0.05	62.80%	There were no significant correlations among plasma norepinephrine, thyroid hormones and RMR.
Konrad 2007	1015.33	1137.33	*P* < 0.05	12.02%	A trend toward smaller increases in RMR being associated with higher admission BMI (*r* =-0.49, *P* = 0.08), but not with highest lifetime BMI. Refeeding is associated with an increase in RMR that is not accounted for by the increase in FFM.
DosReis 2022	864.00	1325.00	*P* = 0.037 Pairwise Kruskal-Wallis test adjusted by bonferroni	53.36%	
**Basal Metabolic Rate (BMR)**					
**Restricting**					
Moukaddem 1997	993.07	1099.67	*P <* 0.021	10.73%	
**Unspecified**					
Forman-Hoffman 2006	1000	1220	Yes	22.00%	Median change: 182 kcal/day, *P* = 0.02
Pichard 1996	969	1360	Not reported	40.35%	
Polito 2000	938.93	1110.08	*P* < 0.002	18.23%	BMR was significantly correlated with FFM (*r*^2^ = 0.48, *P* < 0.0000) and body weight (*r*^2^ = 0.62, *P* < 0.0000). The regression equation showed that 62% of the variance in BMR was attributed to differences in body weight. When BMR was regressed against FFM, 48% of the variance was explained by FFM. A positive correlation was found also between the logarithm of leptin concentration and BMR (*r*^2^ = 0.28, *P* < 0.00006) even after adjustment for FFM (*r*^2^ = 0.21, *P* < 0.0003).

AD: admission; DIS: discharge. LBM: lean body mass; FFM: fat free mass

Seven of the included studies adjusted the measured EE for body weight, lean body mass, or fat-free mass. In four of those studies [33, 38, 54, 55], the EE remained significantly lower at T1 than T2 even after the adjustment. In contrast, the remaining three studies [13, 56, 57] reported that the statistically significant difference diminished after accounting for body weight, lean body mass, or fat-free mass (FFM). One study showed that the ratio of REE to FFM significantly correlated with energy intake, anxiety, abdominal pain, and depressive mood [45]. The ratio also increased significantly with physical activity and cigarette smoking. A positive correlation between serum leptin levels and EE was reported in studies by Haas et al. [58] and Polito et al. [59]. However, no statistically significant correlation was found in a study published a few years earlier by Haas [44].

Twelve of the included studies [37, 38, 43–45, 51, 53, 55, 60–63] used multiple EE values across the renourishment period. Three of the studies [38, 44, 61] included EE measurements at the beginning, middle, and end of treatment, with no significant evidence of a hypermetabolic period during the middle of treatment compared to discharge. Furthermore, Haas 2005 reported that discharge REEs were still lower in AN than their non-ED controls [44]. Similarly, Obarzanek 1994 reported significant increases in RMR when patients were restudied during early refeeding [53]; RMR further increased during late refeeding to levels comparable to healthy volunteers but reverted to values lower than those of controls when the patients finished the target weight-stabilization. Vaisman 1991 and Vaisman 2004 reported gradual increases in REE (across either body weight percentages [63] or treatment duration [55]) persisted until stabilizing near discharge (8–10 weeks). Despite these increases, however, the mean REE failed to reach the “normal” range (90–110% of predicted REE) during hospitalization [55].

In contrast to gradual increases over time, Schebendach 1997, Rigaud 2000, and Pichard 1996 reported that the early weeks of refeeding (i.e., weeks 0–2) are the critical period for statistically significant increases in EE, as higher energy expenditure does not seem to persist in longer-term weight stable patients [37, 51, 62]. Additionally, Van Wymelbeke 2004 reported that the initial increase in REE during the first week of treatment represented 31% of the total REE increase over 2.5 months of refeeding [45]. Importantly, Krahn 1993 and Moukaddem 1997 reported EE results with detailed energy intake information [43, 57]. As patients increased their energy intake to 3600 kcal/day (by 300 kcal/day increments starting at 1200 kcal/day at baseline), Krahn 1993 reported that observed differences in REE ranged from 467 to 1049 kcal/day, highlighting large variability in metabolic recovery [43]. Moukaddem 1997 reported an 8% increase in REE after 1 week of refeeding, with slight variations in estimations based on 300 kcal or 700 kcal experimental loads [57].

### Predicted energy expenditure

Predicted energy expenditure results across two timepoints were extracted if raw values were reported (i.e., not only as percentages of measured EE; Table 3). For consistency within this review, only predictions using the Harris-Benedict equation (*n* = 6) (most widely used [64]) or Bioelectrical Impedance Analysis (BIA; *n* = 1) were extracted. Five of the studies reported statistically significant changes between predicted EE values at baseline and follow up [13, 36, 44, 52, 60], and all studies that included raw predicted EE values reported the finding that predicted values overestimated measured EE in patients with AN. On average, the studies reported a 11.65% change in EE from baseline to follow-up, over 50% less than the average percent change in measured EE. Agüera 2015 reported the smallest percent change of 2.07% in patients with restricting AN [60], and DosReis 2020 reported the largest percent change of 53.36% [35]. Interestingly, Konrad 2007 reported that measured values of EE were 84% below values predicted with the Harris-Benedict Eq.  [38]. These findings are consistent with prior literature suggesting that REE is likely to be higher than predicted during refeeding [13] due to the failure of predictive equations to adequately account for altered body composition and/or hormonal status in females with AN.

**Table 3:** Predicted Energy Expenditure

Study	Method	Baseline EE (kcal/day)	Discharge EE (kcal/day)	Statistical Significance	Percent Change	Comparison to measured and other notes
**Resting Energy Expenditure (REE)**						
**Restricting**						
Melchior 1989	HB equation	1195	1261	*n.s.*	5.52%	Overestimated
Cuerda 2007	HB equation	1263	1307.5	*P* = 0.003	3.52%	Overestimated
**Unspecified**						
Cuerda Compés 2005	HB equation	1271.6	1302.4	*P* < 0.01	2.42%	Overestimated
Haas 2005	HB equation	1261.95	1340.82	*P* < 0.05	6.25%	Overestimated
Kochavi 2020	HB equation	1276.40	1417.90	Not reported	11.09%	Overestimated
**Resting Metabolic Rate (RMR)**						
**Restricting**						
Platte 1994	HB equation	1250	1458	Yes	16.64%	Overestimated
**Basal Metabolic Rate (BMR)**						
**Restricting and Binge eating/Purging**						
Agüera 2015	BIA	ANR:1224.5 BP:1182.6	ANR: 1249.8 BP: 1229.6	Mean differ.[95% CI]: ANR: 25.30 [12.24, 38.26] BP: 47.02 [30.84, 63.21]	ANR: 2.07% BP: 3.97%	Magnitude of change in BMR was significantly greater in BP than in ANR (*P* < 0.001), indicating that pre-post mean changes were different for the two diagnostic conditions.

HB: Harris Benedict; BIA: Bioelectrical impedance analysis; ANR: Restricting Type; BP: Binge-eating and purging type

## Discussion

### Summary of findings

A comprehensive search revealed a complex relationship between metabolic trajectories and the renourishment process in AN, and the included studies reported consistently lower energy expenditure at the initiation of renourishment, compared with values reported in non-eating disorder controls. All studies highlighted subsequent increases in energy expenditure after differing durations of refeeding, with varying levels of statistical significance. The consistent report of moderate increases was not accompanied by reproducible reports of EE-associated clinical variables (e.g., BMI, FFM, or hormones) or a standard duration of refeeding needed to elicit these changes. Additionally, the studies, despite moderate increases, do not indicate evidence of energy expenditure that would indicate the presence of a *hyper*metabolic state, or energy expenditure > 110% of predicted REE [65]. This consensus definition of a hypermetabolic threshold relies heavily on studies of cancer cachexia and burn injuries, and the reliability of applying this numerical value in AN remains questionable due to frequent inaccuracies in predicted REE. Metabolic rates of the patients with AN at the second timepoint (either discharge from treatment, after a short period of refeeding, or recovered individuals) throughout all included studies fall within the normal range (total energy expenditure of 1910–2140 kcal/day for healthy female teenagers [66] and 1572–3687 kcal/day in healthy adults [67]). In summary, weight gain resistance in AN treatment does not seem to be fully explained by measures of metabolic rate and/or energy expenditure. Although there are increases in expenditure after initiation of renourishment, the data suggests that these increases are simply a normalization of expenditure out of the *hypo*metabolic disease state and do not indicate *hyper*metabolism-driven weight gain resistance.

### Limitations of extant literature

Although metabolic adaptations before and after treatment were described, the existing data do not support formal conclusions about metabolic changes that may occur *during* refeeding. Only 33% of included studies (*n* = 12) reported more than two measurements, and many studies did not take the second energy expenditure measurement during the highest energy intake period but rather once the energy intake requirement declined prior to discharge. Moreover, the current literature does not report sufficient data to characterize differences between restricting and binge eating/purging AN subtypes, although one study reported BMR to be higher in the restricting subtype [60]. It is also likely that different measurement techniques for assessing and/or predicting metabolic rate may lead to vastly different estimates, creating an additional layer of difficulty when developing literature-based nutritional rehabilitation programs. Most included studies did not report effect sizes, making the associated clinical relevance hard to interpret. Additionally, metabolic changes in other eating disorders and/or other populations of patients with AN, such as males and/or patients presenting in larger bodies (i.e., atypical AN [68]), are not well described. This warrants future investigation, as males are typically considered to report higher variations in metabolic activity [69] and individuals with higher weights may have significantly different metabolic rates compared to lean individuals [70–72].

Additionally, there is large variability in both length of stay and weight change among the included studies. Three studies with both short (7 days) [42, 57] and long (up to 11 months) [39] length of stay in the unit reported less than 1 unit change in BMI, which complicates the interpretation of these findings. Small weight changes may reflect changes in hydration status more than compartmental changes to body composition. When REE results from these reports are not considered, however, there is still no evidence of hypermetabolic spikes at discharge in the remaining studies. Variations in length of stay, weight change, and comparison groups highlight the need for more consistent characterization of metabolic changes during recovery.

### Increasing scientific rigor

To gain a deeper understanding of the mechanistic basis of increases in energy expenditure in patients with AN during renourishment, it is imperative that the field fosters greater scientific rigor by seeking consistency across design, measurement, and analysis. The included studies discuss multiple hypotheses about why this hypermetabolic phenomenon may occur—such as shifts to bone formation [73], the continuation of protein synthesis in a resting state instead of just after meals [53], dietary induced thermogenesis spikes with greater amount and frequency of food [50]—but explanations for such a prompt rebound to or beyond typical metabolic rates remains elusive, leaving practitioners to adjust energy intake during this period without properly informed evidence-based guidance. Additionally, growing evidence from genetic studies uncovering both psychiatric and metabolic genetic underpinnings to AN [74] adds urgency to designing and conducting rigorous studies to more deeply understand metabolism in individuals with AN, both as a predisposing trait and additionally to understand changes in metabolism over time. As genetics explain ~ 40% of the variance in resting metabolic rate [75] and AN has twin-based heritability estimates of 50–60% [76], investigating the overlap between genetically influenced metabolic traits and AN phenotypes may provide meaningful insight.

Determining whether the purported hypermetabolic phase actually occurs is of considerable clinical importance. Tolerating increasingly high caloric prescriptions is physically and psychologically difficult for patients and can lead to marked gastrointestinal and emotional distress. Moreover, preventing the loss of therapeutically restored weight after discharge depends on accurate prediction of energy requirements to maintain or gain weight. If patients are not gaining weight with a prescribed caloric level and there is no evidence of hypermetabolism, then other reasons for the failure to gain weight must be explored.

Of relevance for refeeding, the primary role—among many—of the intestine is to harvest calories from the diet to sustain the body’s energy requirements. Prolonged caloric restriction can lead to a dysfunctional gut and potentially reduced calorie harvest from the diet [77]. *Indeed, nutrient deprivation in individuals with AN could impact gut function and ultimately lead to a global reduction in the absorptive capacity of the gut, which may be misinterpreted as hypermetabolism during renourishment*. Stated another way, although patients are being fed increasing numbers of calories, their guts may be unable to harvest and utilize those calories for weight restoration. Although renourishment, weight gain, and weight maintenance are major hurdles for recovery, scant information exists about the absorptive capacity of the gut, and its corresponding relationship with metabolism, in patients with AN. The very limited number of studies of intestinal epithelial alterations in AN reported disturbances in tissue architecture and a decrease in intestinal permeability [78, 79], and it is logical to posit that the microbial ecosystem inhabiting the gastrointestinal tract may be an additional barrier [32, 77]. As the body of research progresses, studies with consistent metabolic tracking may lead to investigation of the existence of a dysfunctional intestine in patients with AN, which may have significant implications for developing more effective and enduring renourishment strategies.

### Recommendations

The clinical experience of hypermetabolism, albeit lacking robust empirical evidence, has a direct impact on renourishment protocols implemented by the clinical care team. If a patient is not making expected weight gain, all possible reasons should be explored in order to make informed clinical decisions. Although fear of weight gain may indeed lead patients to engage in treatment-interfering behaviors that inhibit weight gain, it should never automatically be assumed that this is the cause of a weight stall. Careful characterization of energy expenditure throughout the duration of treatment provides the best data for informed decision making among providers. Based on this review, considerable research is needed to enrich our understanding of metabolic factors and changes that are relevant to AN etiology, progression, treatment, and remission. As a start, we recommend the following:

I. Future research should harmonize methodologies and standardize reporting of results. Robust studies should include detailed dietary data (i.e., energy intake), subtype categorizations, and information about the duration of illness. In addition to significance testing, effect sizes should be reported as well as metrics that capture the extent to which results are clinically meaningful.
II. If physiological measurement via indirect calorimetry, doubly labeled water, or other methods is not available, studies utilizing predictive equations for energy expenditure should include subtype, body composition measures (fat free mass and fat mass through dual X-ray absorptiometry or bioelectrical impedance analysis [80]), and duration of illness in the equations for better predictive accuracy [15]. Recent findings from Bou Khalil et al. report that if FFM and FM measurements are not available, the Schebendach equation [81] will have the highest agreement with REE measured by indirect calorimetry [15]. Authors employing these methods should highlight shortcomings of these equations; for example, Cuerda 2007 reported that measured REE values did increase throughout hospitalization but still remained 10% lower than predicted values [13]. Treatment teams in the United States are frequently faced with the challenge of discharge prior to weight stabilization despite recent evidence that BMI at the end of treatment is a direct predictor of relapse [82]. In a climate of insurance thresholds that are based on percentages—such as discharge at 80% ideal body weight—it is imperative to acknowledge the implications of inaccurate expenditure predictions on weight stabilization and relapse risk. Although further research is needed to establish the most clinically relevant timepoints for energy expenditure assessment, we recommend energy expenditure estimations at admission, approximately two weeks into treatment, and prior to discharge to inform dietary prescriptions to support continued weight gain or weight maintenance. Although duration of inpatient stay is difficult to predict and varies due to many factors, a second measurement after a week or two of treatment will capture energy expenditure once fluid and electrolyte levels have stabilized. It is possible that persistent disruptions in homeostatic energy expenditure, in the presence of other indicators of active illness (such as lack of weight stabilization) may be important factors to consider prior to discharge at a certain percentage of ideal body weight.
III.  Funding for well-developed studies with a comprehensive characterization of energy expenditure over time, including multiple timepoints during renourishment, is imperative. Additionally, funding to investigate biological mechanisms that may be contributing to difficulties during treatment will greatly advance the field. Relevant biological avenues for exploration include: (i) the intestinal microbiota (including the development of microbiota directed complementary foods to be implemented during refeeding), (ii) genetics (with metabolic-, psychiatric- and nutri-genetic driven hypotheses), (iii) structural or physiological changes to the gut in AN, and (iv) other feeding and eating behaviors, such as time restricted eating, binge eating, and purging, that may play a role in the absorption or conversion of calories to tissue.

## Conclusion

This scoping review provides an updated description of research examining variations in energy expenditure before and following a period of renourishment in patients with AN. After summarizing reports of measured and predicted EE, it is clear that patients currently ill with active AN present for treatment with slowed metabolic rates, and, after treatment, experience mild to moderate increases in metabolic rate. The commonly occurring and resolute resistance to weight gain, however, cannot be fully attributed to a hypermetabolic shift during renourishment. This review has highlighted many gaps for which further funding and research is essential, as there is an urgent need to explore metabolic abnormalities in AN. The reality faced by patients with AN is often a bleak one—with approximately one third of individuals with AN still not reaching recovery after 22 years of illness [83]. Decades of research into psychological and pharmacological interventions have left all those in the AN field—patients, caregivers, providers, and researchers alike—in need of more viable treatment options that provide a more promising future [84]. In a continued effort to shift the treatment paradigm, we propose that now is the time to finally understand the perplexing and remarkably resilient metabolic adaptions that occur in AN.

## Data Availability

No datasets were generated or analysed during the current study.

## Abbreviations

AN: anorexia nervosa
Kcal: kilocalorie
ATP: adenosine triphosphate
BMR: basal metabolic rate
EE: energy expenditure
REE: resting energy expenditure
TEE: total energy expenditure
RMR: resting metabolic rate
PRISMA: Preferred Reporting Items for Systematic Reviews
DSM: Diagnostic and Statistical Manual of Mental Disorders
ED: eating disorder
T1: timepoint 1
T2: timepoint 2
BMI: body mass index
FFM: fat free mass
